# Leisure Barriers Among Older Adults in Low-Income Housing: Demographic, Health, and Contextual Correlates

**DOI:** 10.1093/geroni/igaa057.1920

**Published:** 2020-12-16

**Authors:** Angela Sardina, Shyuan Ching Tan, Alyssa Gamaldo

**Affiliations:** 1 University of North Carolina Wilmington, Wilmington, North Carolina, United States; 2 Penn State University, State College, Pennsylvania, United States; 3 Penn State, University Park, Pennsylvania, United States

## Abstract

Despite increased research pertaining to the physical, cognitive, and psychosocial benefits of leisure engagement, few studies have explored leisure barriers experienced by older adults residing in subsidized housing, and how these barriers relate to sociodemographic, health, and psychosocial characteristics. Thirty-nine Black and White residents (M=68.01, SD=10.26) from two subsidized housing communities (Wilmington, NC and State College, PA) were surveyed as part of the Tailoring Environments for Active Life Engagement study. Findings indicated that lack of available activities and low awareness of activities, limited social connections, and transportation were the most common barriers identified. Additionally, individuals with lesser years of education and poorer quality of education, worse mental and physical health, poorer cognitive function, as well as those experiencing loneliness and social isolation reported significantly more leisure barriers (ps <.05). More research is needed that examines micro-, meso-, and macro-level factors associated with leisure participation for older low-income housing residents.

